# α-synuclein oligomers interact with ATP synthase and open the permeability transition pore in Parkinson’s disease

**DOI:** 10.1038/s41467-018-04422-2

**Published:** 2018-06-12

**Authors:** Marthe H. R. Ludtmann, Plamena R. Angelova, Mathew H. Horrocks, Minee L. Choi, Margarida Rodrigues, Artyom Y. Baev, Alexey V. Berezhnov, Zhi Yao, Daniel Little, Blerida Banushi, Afnan Saleh Al-Menhali, Rohan T. Ranasinghe, Daniel R. Whiten, Ratsuda Yapom, Karamjit Singh Dolt, Michael J. Devine, Paul Gissen, Tilo Kunath, Morana Jaganjac, Evgeny V. Pavlov, David Klenerman, Andrey Y. Abramov, Sonia Gandhi

**Affiliations:** 1 Department of Molecular Neuroscience, UCL Institute of Neurology, London, WC1N 3BG UK; 2 Royal Veterinary College, 4 Royal College St, Kings Cross, London, NW1 0TU UK; 3 Department of Chemistry, University of Cambridge, Lensfield Road, Cambridge, CB2 1EW UK; 4 EaStCHEM School of Chemistry, University of Edinburgh, David Brewster Road, Edinburgh, EH9 3FJ UK; 5 UK Dementia Research Institute, University of Edinburgh, Edinburgh, UK; 6 Sobell Department of Motor Neuroscience and Movement Disorders, UCL Institute of Neurology, Queen Square, London, WC1N 3BG UK; 7 The Francis Crick Institute, 1 Midland Road, King’s Cross, London, NW1 1AT UK; 8 Educational-Experimental Centre of High Technologies, Laboratory of Biophysics and Biochemistry, Tashkent, Uzbekistan; 9 Institute of Cell Biophysics, Russian Academy of Sciences, Pushchino, 142290 Russia; 10 MRC Laboratory for Molecular Cell Biology, University College London, Gower Street, London, WC1E 6BT UK; 11 Toxicology and Multipurpose Department, Anti-Doping Lab Qatar, Sport City Road, PO Box 27775, Doha, Qatar; 12 MRC Centre for Regenerative Medicine, Institute for Stem Cell Research, School of Biological Sciences, The University of Edinburgh, Edinburgh, EH16 4UU UK; 13 Department of Neuroscience, Physiology and Pharmacology, University College London, London, WC1E 6BT UK; 14 Department of Basic Sciences, New York University College of Dentistry, NY, 10010 USA; 15 UK Dementia Research Institute, University of Cambridge, Cambridge, CB2 0XY UK

## Abstract

Protein aggregation causes α-synuclein to switch from its physiological role to a pathological toxic gain of function. Under physiological conditions, monomeric α-synuclein improves ATP synthase efficiency. Here, we report that aggregation of monomers generates beta sheet-rich oligomers that localise to the mitochondria in close proximity to several mitochondrial proteins including ATP synthase. Oligomeric α-synuclein impairs complex I-dependent respiration. Oligomers induce selective oxidation of the ATP synthase beta subunit and mitochondrial lipid peroxidation. These oxidation events increase the probability of permeability transition pore (PTP) opening, triggering mitochondrial swelling, and ultimately cell death. Notably, inhibition of oligomer-induced oxidation prevents the pathological induction of PTP. Inducible pluripotent stem cells (iPSC)-derived neurons bearing *SNCA* triplication, generate α-synuclein aggregates that interact with the ATP synthase and induce PTP opening, leading to neuronal death. This study shows how the transition of α-synuclein from its monomeric to oligomeric structure alters its functional consequences in Parkinson’s disease.

## Abstract (web summary)

How toxic aggregated forms of α-synuclein lead to neurodegeneration is unclear. Here authors use biophysical and cellular imaging methods to show that specific oligomers of α-synuclein exert effects on mitochondria to induce opening of the permeability transition pore, leading to cell death in Parkinson’s disease.

## Introduction

Protein aggregation and mitochondrial dysfunction are two central pathogenic processes in both familial and sporadic Parkinson’s disease (PD). However, the way in which these two processes converge to cause neurodegeneration is unknown. Missense mutations^1,2^, and duplications or triplications of the *SNCA* gene, which encodes α-synuclein, lead to autosomal dominant early-onset PD^3,4^, that is clinically and pathologically similar to sporadic PD. Genetic and biochemical data suggest that, as the concentration of α-synuclein increases, especially in the presence of high levels of dopamine^5^, the tendency for it to self-aggregate and form oligomers, and eventually fibrils also increases^6^. Aggregation of α-synuclein from its monomeric to oligomeric form leads to structural conformation changes in the protein that mediate the toxic effects of α-synuclein within cells^7–9^.

We recently reported that α-synuclein, in its monomeric state, interacts with, and regulates ATP synthase to improve the efficiency of ATP production^10^. Here we investigate whether the mitochondrial function of α-synuclein is structure-specific, and therefore adopted a range of methods including single-molecule biophysical measurements, super-resolution microscopy, electrophysiology, and dynamic fluorescent neuronal imaging to describe the location and functional mechanism of oligomeric species of α-synuclein, compared to monomeric species. We show that α-synuclein oligomers come into close proximity with, and exert, functional effects on several mitochondrial proteins. This study focuses on its specific interaction with ATP synthase due to the previously reported physiological interaction with α-synuclein, and the putative role of ATP synthase in the regulation and/or composition of the PTP.

## Results

### Generation and characterisation of aggregated α-synuclein

**Fig. 1:**
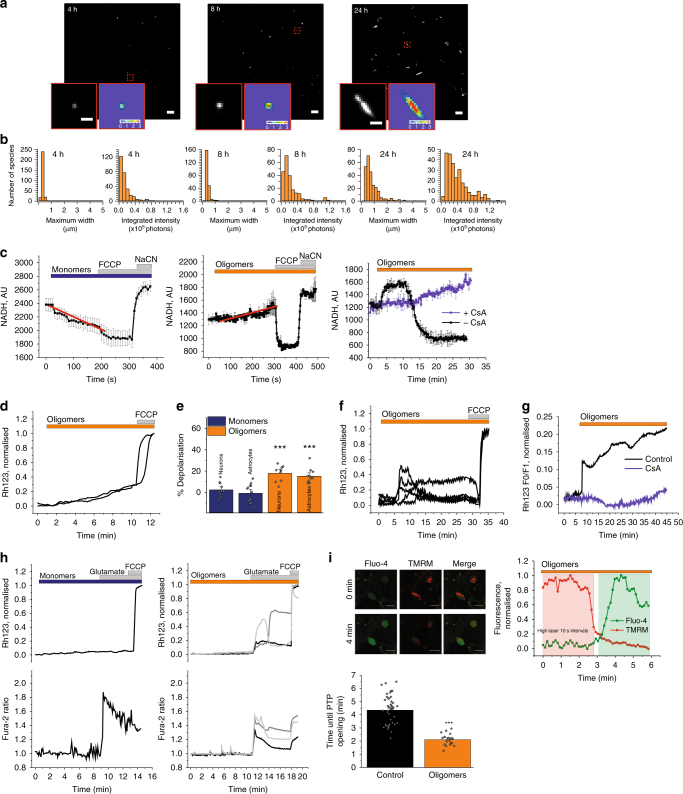
Characterisation of oligomers and their effect on mitochondria. **a** Representative SAVE images of early oligomers (4 h), late oligomers (8 h), and fibrils (24 h). Zoom and representative two-dimensional Gaussian distribution fits are shown in the insets. The scale bar is 5 µm and 1 µm in the zoom, and the colour bar shows the Gaussian amplitude (×10^4^ photons). **b** Quantification of aggregation. Each detected species was fitted to a two-dimensional Gaussian distribution, and histograms of the widths (FWHM) along the longest axis, and the total integrated intensities are shown for each time-point. **c** Representative traces from single experiments, of NADH autofluorescence in WT neurons exposed to either monomers (*n* = 15 cells), oligomers (*n* = 32 cells), or oligomers ± CsA (*n* = 9 cells and *n* = 6 cells, respectively). **d** Representative Rh123 traces of cells challenged with oligomers. **e** Quantification of mitochondrial depolarisation upon either monomeric or oligomeric application. *N* = 3 experiments; Oligomers: *n* ≥ 60 neurons/astrocytes; Monomers: *n* ≥ 50 neurons/astrocytes. **f** Representative Rh123 traces of cells challenged with oligomers. **g** Representative Rh123 traces of cells challenged with oligomers ± CsA. **h** Representative traces of Rh123 and fura-2 in WT neurons exposed to glutamate in the presence of monomers or oligomers. **i** Representative images of WT rat neurons labelled with fluo-4 (cytosolic calcium) and TMRM (ΔΨm) before (0 min) and after (4 min) high laser exposure which represents PTP opening. A representative trace of TMRM and Fluo-4 fluorescence where the drop in TMRM fluorescence precedes increase in Fluo-4 fluorescence. Quantification of the time until PTP opening in WT cells pre-exposed to oligomeric α-synuclein. *N* = 3 experiments; *n* = 43 cells for control and *n* = 20 cells for oligomers. Two-tailed Student’s *t*-test for **e** and **i**. Scatter points represent individual cells for **e** and **i**. Scale bar = 10 μm. Data represented as mean ± SEM. *p < 0.05; **p < 0.01; ***p < 0.001

We generated aggregated forms of recombinant α-synuclein that were characterised using a highly sensitive single-molecule method termed Single Aggregate Visualisation by Enhancement (SAVE) imaging, which uses single-molecule fluorescence microscopy to detect the benzothiazole salt Thioflavin-T (ThT)^11^. Upon binding to β-sheet structures, ThT fluorescence increases allowing individual aggregated species to be detected (Fig. 1a). This can be fitted to a two-dimensional Gaussian distribution to determine the aggregate width along the longest axis (full-width half maximum), in addition to the integrated intensity of each species (Fig. 1b). From 2 h onwards, the number of diffraction limited fluorescent puncta increases, which represents the emergence of oligomers. At later time points (24 h), fibrils as long as 5 µm are observed. The total integrated intensity also increases, representing the increased crossed β-sheet content in the larger aggregates (Fig. 1b).

For this study, we utilised time points characteristically represented by Fig. 1a (8 h), in which ~1% of the aggregation mixture has formed β-sheet-rich oligomeric structures, and the remaining 99% is monomeric. As a paired control to the oligomer preparation, monomer sample from the starting time point of the aggregation was used.

### Oligomers induce mitochondrial dysfunction in whole cells

To investigate the effect of structural conformation on mitochondrial function, monomers and oligomers were applied to primary rat co-cultures of neurons and astrocytes and the autofluorescence of mitochondrial NADH (selected from NADH/NADPH autofluorescence determined by activation and inhibition of NADH consumption—see Fig. 1c) was assessed. 100 nM monomeric α-synuclein induced a decrease in NADH autofluorescence in wildtype (WT) neurons (Fig. 1c) suggesting consumption of NADH through activation of respiration. In contrast, oligomers (100 nM monomers, ~1 nM oligomers) induced a slow and progressive increase of NADH in mitochondria (Fig. 1c) confirming inhibition of complex I by oligomeric α-synuclein (in agreement with previous reports)^12^. However, more prolonged exposure of cells with oligomers, in some neurons, induced a sudden drop in mitochondrial NADH, which could be induced by rapid activation of NADH consumption due to uncoupling of mitochondria or by mitochondrial permeability transition pore (PTP) opening and redistribution of NADH to cytosol (Fig. 1c). Inhibitor of mPTP cyclosporin A (CsA) prevented this transient drop in mitochondrial NADH level (Fig. 1c). Impairment of complex I function may affect the mitochondrial membrane potential (ΔΨm). Application of oligomers (~1 nM oligomers) induced slow mitochondrial depolarisation, reflected in the slow increase in Rh123 intensity (Fig. 1d, e). This gradual depolarisation of the ΔΨm is in accordance with oligomer-induced impairment of respiration and reduced electron flow through the electron transport chain. Monomers alone did not induce mitochondrial depolarisation, and may even hyperpolarise mitochondria, as previously shown^10^.

Application of oligomers (~3 nM oligomers) also induced sharp transient increases in Rh123 fluorescence intensity, which appeared as discrete mitochondrial depolarisation events that resembled PTP opening (Fig. 1f). When cells were pre-incubated with the PTP inhibitor cyclosporine A, the oligomer-induced PTP-like events were abolished (Fig. 1g).

PTP opening in mitochondria can be triggered by two major stimuli, calcium and reactive oxygen species (ROS). First we tested whether a physiological calcium stimulus, low concentration of glutamate (1 μM), could trigger PTP opening in the presence of oligomers (~1 nM oligomers). Application of glutamate to WT rat neurons leads to a rapid cytosolic calcium influx with no significant alteration in the basal ΔΨm (Supplementary Fig. 1). Incubation of WT rat neurons with monomers for 6–9 min prior to the addition of glutamate-induced a similar cytosolic calcium and ΔΨm response to control (Fig. 1h). Conversely, exposure to oligomeric α-synuclein prior to glutamate stimulation resulted in a glutamate-induced cytosolic calcium response that was associated with rapid and transient depolarisation with incomplete recovery to baseline, which resembled PTP opening (Fig. 1h). Next, we assessed whether oligomers altered the PTP threshold in response to laser-induced ROS production. Cells loaded with fluo-4 and tetramethylrhodamine methyl ester (TMRM) (ΔΨm; Fig. 1i), were treated with oligomers and then exposed to high laser power. High laser power generates ROS, which in turn triggers PTP opening that leads to a rapid mitochondrial depolarisation reflected by a rapid fall in TMRM signal (Fig. 1i). This is then followed by an increase in the cytosolic fluo-4 fluorescence as the mitochondrial calcium redistributes to the cytosol (note the appearance of TMRM drop before fluo-4 increase; Fig. 1i). The threshold for PTP opening was significantly lower in oligomer-treated cells when compared to control conditions (HBSS only, *p* *<* 0.001; Fig. 1i) suggesting that oligomers lower the threshold for ROS-induced PTP opening in neuronal co-cultures.

### Oligomers induce PTP opening in isolated mitochondria

In order to determine whether PTP opening was a direct effect of oligomers within mitochondria, or a downstream effect of secondary cellular pathways, the effect of α-synuclein on isolated mitochondria was investigated.

**Fig. 2:**
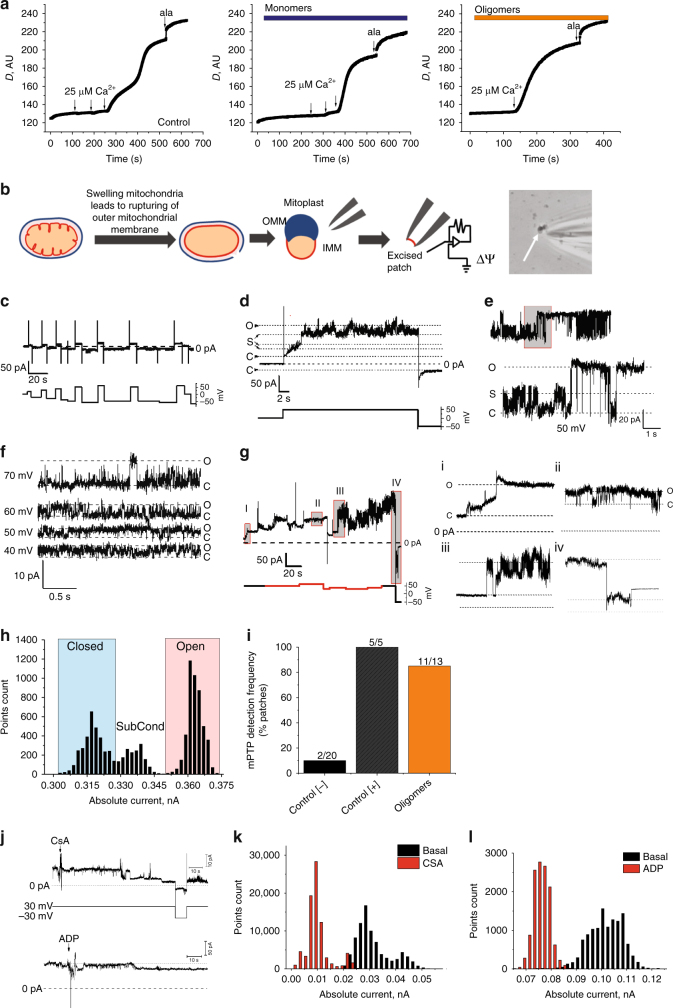
Oligomers but not monomers induce PTP opening. **a** Mitochondrial swelling was measured by changes in optical density (*D*) in the presence of buffer, monomers, and oligomers. Complete mitochondrial swelling was achieved by the addition of 5 μM alamethicin. **b** Schematic diagram of mitoplast preparation and patch-clamp experiment. **c** Patch clamp recording of the inner membrane from control mitoplasts prepared in calcium-free media in the absence of mitochondrial energy substrates. **d** Patch clamp recording of the inner membrane from control mitoplasts prepared in calcium media in the presence of succinate showing activation of PTP. **e** Typical multi-conductance state of single PTP channel, induced by calcium (positive control). **f** Novel channel activity detected in the presence of α-synuclein oligomers in the pipette solution, other conditions the same as in **a**. **g** Oligomer-induced gradual development of channel activity (i and ii), which later transformed into full conductance channel resembling PTP (iii) which was inactivated/closed by the application of high negative potential (iv). **h** Current amplitude histogram of the channel shown in **e**. **i** Detection frequency of the PTP channel in different experimental conditions. **j** Effects of PTP inhibitors CSA (1 µM) and ADP (1 mM) on channel activity. **k**, **l** Current amplitude histogram of the channel shown in **j**

First, a swelling assay on isolated mitochondria was performed^13^, in which mitochondrial swelling was estimated from the kinetics of the intensity of the transmitted light. Monomers (100 nM) or oligomers (100 nM monomer, ~1 nM oligomers) were applied to energised mitochondria for 3–5 min, followed by stepwise additions of calcium applied to trigger PTP opening. In the presence of monomers, PTP opening and mitochondrial swelling were triggered after three additions of 25 μM calcium (Fig. 2a), whereas oligomers triggered opening after the first addition of 25 µM calcium.

Next, we employed mitochondrial electrophysiology to characterise the alteration in membrane conductance that occurs across the inner mitochondrial membrane in response to α-synuclein. Mitoplasts were prepared from isolated mitochondria and patch-clamp experiments were performed (Fig. 2b). Negative control mitoplasts were prepared by passive swelling in substrate-free and calcium-free media. Positive control mitoplasts were prepared by swelling induced by calcium-treatment of energised mitochondria to activate PTP. As expected, mitoplasts prepared in the absence of calcium exhibited little or no ion channel activity (Fig. 2c), while calcium-treated mitochondria demonstrated high conductance channel activity (Fig. 2d, e), with properties resembling the multiple conductance channel or PTP characterised by an averaged peak conductance of ~3.6 nS. Channel activity induced by α-synuclein was compared to channel activity seen in calcium-treated mitoplasts.

When oligomers (~1–3 nM oligomers; 100–300 nM monomers) were present in the pipette, all patches showed discrete ion channel activity (average transition size of 0.46 ± 0.15 nS), not seen in control (Fig. 2f for typical single channel behaviour and Table 1). Furthermore, in 85% of these oligomer-treated patches (Fig. 2i), channel activity was then followed, within 10 min, by the opening of a megachannel (averaged peak conductance 1.9 ± 0.4 nS) (Fig. 2g and inset i–iv; Table 1) with the properties resembling the PTP channel as seen in our control experiments in the presence of calcium (Fig. 2d, e, h). Channel activity induced by oligomers was inhibited by CsA and partially inhibited by ADP further confirming its relationship to PTP (Fig. 2j–l). Altogether, the detection frequency of PTP-like activity in the presence of oligomers was comparable to the detection frequency of Ca^2+^-induced PTP-like channel, and increased when compared to channel activity seen in the absence of calcium (Fig. 2i).

**Table 1:** Alpha-synuclein-induced channel activity and PTP opening in mitochondrial inner membrane patches

	[−] Control	[+] Control	Monomers	Oligomers	Oligomers + Trolox (in the pipette)
IMM basal membrane conductance [nS]	1.26±0.28	0.24±0.02	0.29±0.12	1.6±0.5	0.52±0.2
Channel occurrence, % of total recordings	27	50	50	100	0
Channel predominant transition size [nS]	0.31±0.16	0.75±0.53	0.46±0.26	0.46±0.15	N/A
PTP occurrence, % of total recordings	10	100	25	85	0
PTP peak conductance [nS]	2.5±1.5	3.1±0.8	1.3±1.3	1.9±0.4	N/A
Time until onset of PTP development [min]	3	1–3	5	9	N/A

IMM: inner mitochondrial membrane; PTP: permeability transition pore

Monomers (300 nM) induced small occasional channels, and rarely induced a megachannel that exhibited a lower peak conductance to the positive control calcium-induced PTP or oligomer-induced PTP (Table 1). Oligomer-induced channel activity and oligomer-induced PTP were not detected in the presence of the antioxidant Trolox in the pipette.

### Oligomers interact with the mitochondrial ATP synthase

We previously demonstrated that monomeric α-synuclein interacts with ATP synthase using co-immunoprecipitation and proximity ligation assay (PLA). Here we investigated whether oligomeric α-synuclein similarly interacts with ATP synthase. To distinguish monomeric from oligomeric α-synuclein within cells, we utilised a conformation-specific filament antibody, raised against aggregated α-synuclein.

**Fig. 3:**
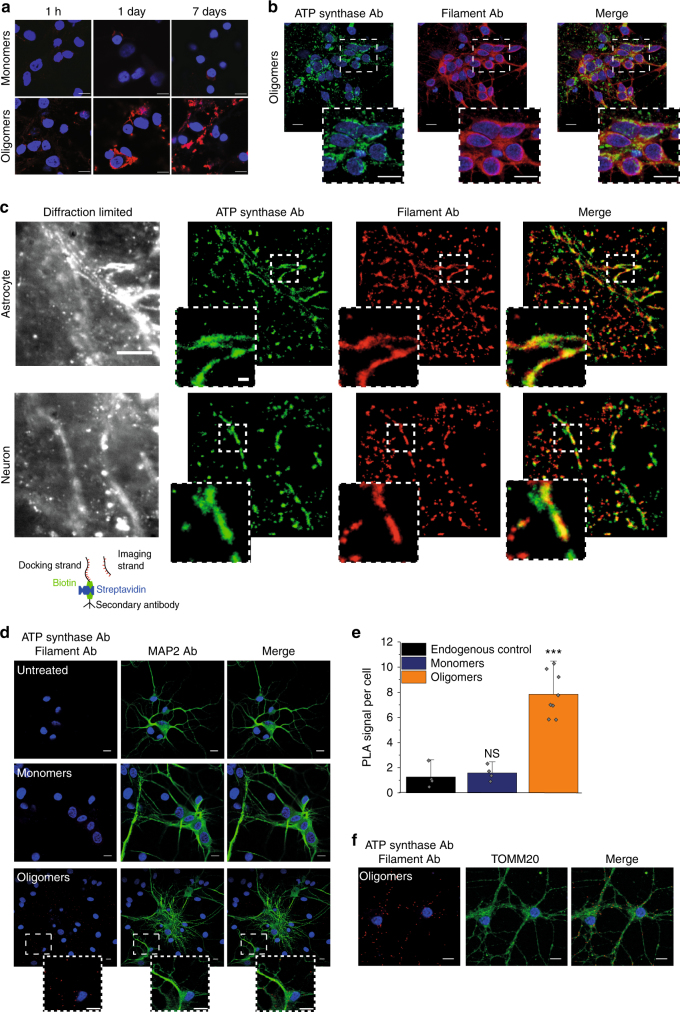
Oligomers co-localise with ATP synthase. **a** Representative images of rat neuronal co-cultures treated with either monomers or oligomers over 7 days and probed with a filament specific antibody. The nucleus can be seen in blue (DAPI). **b** Representative immunocytochemistry images of rat neuronal co-cultures treated with oligomers. The cells were probed for ATP synthase subunit-α and filament α-synuclein. The nucleus can be seen in blue (DAPI). Scale bar = 10 μm. **c** Left: diffraction limited image of α-synuclein (scale bar = 5 µm). Right: super-resolved images of α-synuclein and ATP synthase and merged imaged (scale bars are 500 nm). Schematic diagram of DNA-PAINT: The secondary antibody conjugated with a docking strand binds the primary antibody. A fluorophore labelled complementary imaging DNA strand binds the docking strand on the secondary antibody allowing super-resolution imaging. **d** Representative PLA images of cells treated with either monomers, oligomers or no synuclein probing for ATP synthase subunit α and filament α-synuclein. Cultures were counterstained with the neuronal marker, MAP2. Scale bar = 10 μm. **e** Quantification of PLA signals per cell. *N* = 3 experiments. Oligomers: *n* = 182 cells; Monomers: *n* = 117; Control: *n* = 134 cells. **f** Representative PLA images of cells treated with oligomers probing for ATP synthase subunit α and filament α-synuclein. TOMM20 was probed to validate the mitochondrial localisation of the PLA signal. Two-tailed Student’s *t*-test for **e**. Scatter points represent number of puncta per cell. Data represented as mean ± SEM. ***p < 0.001

Due to the difficulty in preserving aggregate conformation with biochemical methods, we adopted visualisation methods to study at high precision the location and proximity of the α-synuclein aggregate to the mitochondrial protein. The specificity of the antibody for oligomers was tested by exposing WT rat co-cultures to either 500 nM monomers or 500 nM oligomers (500 nM monomers, ~5 nM oligomers) and performing immunocytochemistry (Fig. 3a lower panel). In cells with exogenously added oligomers, a signal could be detected 1 h following addition of oligomers. After 1 day, and at 7 days, there was a significant increase in the signal intensity and appearance of protein aggregates within the oligomer-treated cells, reflecting the potent ability of the oligomers to seed aggregation in the host cell^14^ and for such aggregates to be detected by the antibody. Monomer-treated cells did not show a significant signal using the filament Ab (Fig. 3a upper panel).

Double immuno-labelling using the filament α-synuclein antibody and an antibody raised against ATP synthase subunit α in WT rat co-cultures exposed to oligomers (Fig. 3b) demonstrated areas of co-localisation of aggregated α-synuclein and the ATP synthase. To overcome the diffraction limit of conventional confocal microscopy, super-resolution microscopy was employed^11,15^. DNA-PAINT utilises fluorophore labelled DNA imaging strands that transiently bind to their complementary docking strands that are conjugated to secondary antibodies (see schematic diagram, Fig. 3c). We used two different docking strand sequences with their complementary imaging strand pairs labelled with either ATTO655 or CY3B to simultaneously image α-synuclein aggregates and ATP synthase, respectively. Due to the high photon numbers from these fluorophores, we achieved a precision of 28 nm and 29 nm for ATP synthase and α-synuclein, and because each antibody could be localised multiple times using the PAINT methodology, we measured more than 500,000 localisations in each channel, achieving a resolution of 45–108 nm and 46 nm for α-synuclein and ATP synthase, respectively. At this resolution, there were clear areas within the cell in which aggregates of α-synuclein are co-localised with the ATP synthase (Fig. 3c). To further quantify this, we determined the association quotient, *Q*, for the ATP synthase and α-synuclein filament localisations, which is a measure of the coincidence between the images (see Methods). This was determined to be 0.48. Similar experiments were performed with antibodies to an outer mitochondrial protein, TOMM20, and an inner mitochondrial membrane protein, Complex 1 (resolution = 154 nm and 58 nm, respectively). Regions of co-localisation were also observed between α-synuclein and inner and outer membrane proteins (co-incidence coefficients of 0.31 for TOMM20; 0.62 for Complex 1) reflecting the reported potential interactions of α-synuclein with complex 1 and TOMM20 in the literature (Supplementary Fig. 2).

To further explore a potential interaction between aggregated α-synuclein and ATP synthase, in situ proximity ligation assay (PLA; Fig. 3d) was utilised. 500 nM monomers, oligomers (500 nM monomers, ~5 nM oligomers), or buffer were applied to rat co-cultures and a PLA signal between the antibodies against filament α-synuclein and ATP synthase subunit α was observed, confirming an interaction between aggregated α-synuclein and ATP synthase. Microtubule-associated protein 2 (MAP2) staining was used to identify neuronal cells. Oligomer-treated cells showed a significantly increased interaction between the oligomers and ATP synthase compared to the equivalent concentration of monomers only or control preparation (*p* < 0.01; Fig. 3e). The mitochondrial protein, TOMM20, was probed for to validate the mitochondrial localisation of the PLA signal (Fig. 3f). Further, a PLA was carried out to probe for complex I and filament α-synuclein to act as a positive control for an interaction of α-synuclein with another mitochondrial protein (Supplementary Fig. 3A). As a negative control a PLA between the nuclear histone H3 and filament α-synuclein was used (Supplementary Fig. 3B, C).

### Oligomers induce non-enzymatic ROS production

**Fig. 4:**
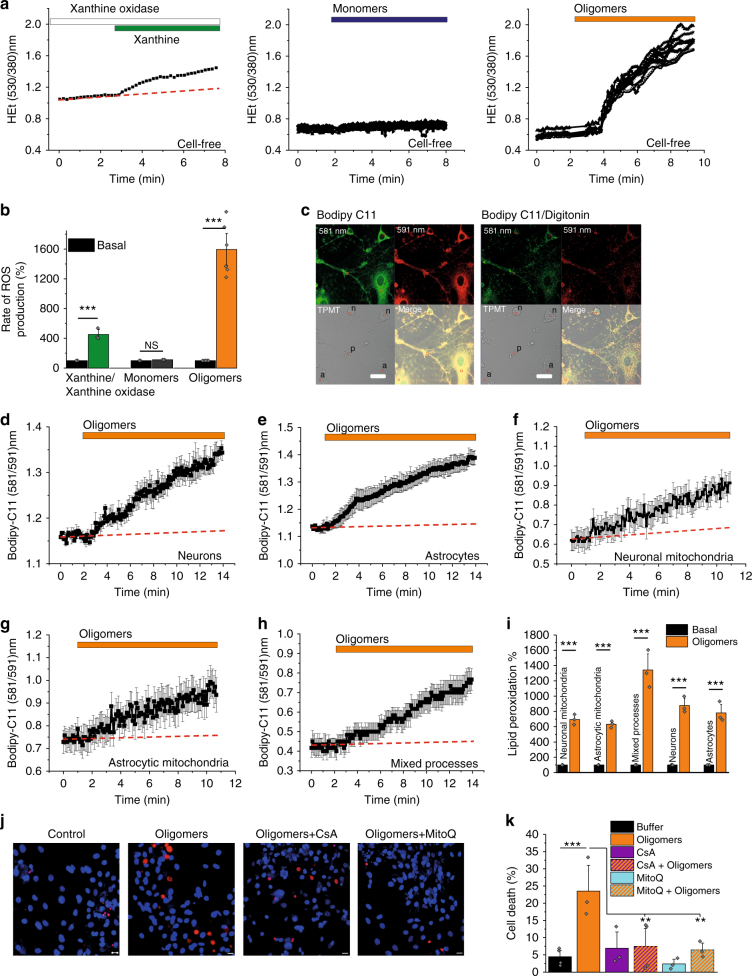
Oligomeric α-synuclein generates ROS and induces lipid peroxidation. **a** Representative trace of ROS generation by xanthine oxidase/xanthine (*N* = 3 experiments; *n* = 14 regions), monomers (*N* = 3 experiments; *n* = 10 regions), and oligomers (*N* = 6 experiments; *n* = 24 regions) in a cell-free system. **b** Quantitative analysis of the cell-free DHE oxidation. **c** Representative images of C11-BODIPY 581/591 labelled cells and areas analysed: (n) neurons, (a) astrocytes, and (p) processes before and after permeabilization with 30 µM Digitonin. **d** Averaged traces of oligomer-induced lipid peroxidation of neurons and **e** astrocytes before permeabilization. **f** Averaged traces of lipid peroxidation after permeabilisation of mitochondria in neurons (**f**), astrocytes (**g**), and mixed processes (**h**). **i** Quantitative analysis of the data of neurons (*N* = 3 experiments; *n* = 26 cells), astrocytes (*N* = 3 experiments; *n* = 16 cells), neuronal mitochondria (*N* = 3 experiments; *n* = 21 regions), astrocytic mitochondria (*N* = 3 experiments; *n* = 20 regions), and mixed processes mitochondria (*N* = 3 experiments; *n* = 28 regions). **j** Representative images and **k** cell death quantification of cells treated with either control buffer, oligomers (*n* = 264 cells), oligomers + CsA (*n* = 189 cells), or oligomers + MitoQ (*n* = 258 cells). *N* = 3 experiments. Two-tailed Student’s *t*-test (**b, i**). One-way ANOVA with Bonferroni correction (**f**) and scatter points represent sampled fields of view (**k**). Data represented as mean ± SEM. Scale bar = 10μm. *p < 0.05; **p < 0.01; ***p < 0.001

Oligomers are potent inducers of cellular ROS^7^, and we hypothesised that this contributes to the oligomer-specific effects. To test whether aggregates of α-synuclein are intrinsically redox-active, we employed a cell-free method of testing oxidation which relies on dihydroethidium (DHE) oxidation after application of α-synuclein^16^. Xanthine/xanthine oxidase ROS production was used as a positive control (Fig. 4a, b). Application of 300 nM monomers (Fig. 4a, b) did not induce any significant oxidation of DHE. However, application of oligomers (300 nM monomers, ~3 nM oligomers) induced oxidation of DHE in the absence of cells (Fig. 4a, b).

Previously we have shown that oligomers could induce lipid peroxidation^17^. Oligomers may be able to induce targeted oxidation at the site of their action, that is, in the vicinity of ATP synthase at the inner mitochondrial membrane. Membrane lipid peroxidation was tested using C11-BODIPY 581/591, (Fig. 4c). An increase in lipid peroxidation following application of oligomers (500 nM monomers, ~5 nM oligomers) in intact neurons and astrocytes was observed, in agreement with previous findings^15^ (Fig. 4d, e, i). Next, C11-BODIPY 581/591-loaded cells were permeabilized using digitonin to isolate the mitochondria, allowing an assessment of mitochondrial lipid peroxidation (Fig. 4c). Application of oligomers to permeabilized cells induced lipid peroxidation in mitochondrial membranes of neurons, astrocytes, and mixed processes (Fig. 4f–i).

Taken together, oligomers generate ROS as an intrinsic property of the oligomer, and are able to oxidise lipid membranes at their site of action, in particular at the inner mitochondrial membrane.

### Oligomeric α-synuclein oxidises ATP synthase subunits

Excessive production of ROS can damage lipid, proteins, and other molecules. The formation of extremely harmful ROS can mediate direct oxidation of proteins^18,19^. In particular, hydroxyl radical readily oxidises sulphur-containing amino acids like methionine and cysteine, thus altering protein conformation and function. Using redox proteomics, we investigated oxidative posttranslational modifications (PTMs) of the ATP synthase in mitochondrial fractions treated with either monomers or oligomers. The obtained data revealed significant oligomer-dependent oxidative modifications of ATP synthase subunit β but not the ATP synthase subunit α, and selected other mitochondrial proteins shown for comparison. (Table 2). Treatment with 100–500 nM α-synuclein (~1–5 nM oligomer) led to a 6–8-fold increase in the number of oxidised amino acids compared to control (untreated) samples, while treatment with 100–500 nM monomer only led to a 3–4-fold increase in oxidised amino acids compared to untreated (Table 2; Supplementary Table 1). To quantify these effects, we immunoprecipitated the ATP synthase from the isolated mitochondria fractions, and then analysed the ratio of peptides with oxidised methionine vs non-oxidised ([Met(O)]/[Met]) peptides. This analysis revealed a highly significant increase of some Met(O) peptides compared to Met peptides of ATP synthase subunit β on oligomer treatment. A representative data of the ratio of four such peptides, one strongly affected by treatment with oligomers (SLQDIIAILGMDELSEEDKLTVSR), and the others not significantly altered is shown in Supplementary Fig. 6.

**Table 2:** Oxidation of amino acids by treatment with α-synuclein

Accession	Description	Gene	ΣCoverage					Σ# Unique Peptides					Σ# Modified AA					# AAs	MW [kDa]
			C	100 M	100 O	500 M	500 O	C	100 M	100 O	500 M	500 O	C	100 M	100 O	500 M	500 O		
G3V6D3	ATP synthase subunit beta	Atp5b	58.22	61.81	50.47	55.95	58.22	19	20	16	18	19	1	3	6	4	8	529	56.3
F1LP05/P15999	ATP synthase subunit alpha	Atp5a1	36.89	41.95	39.78	37.79	39.42	14	14	14	12	13	2	3	0	2	3	553	59.8
P04636	Malate dehydrogenase, mitochondrial	Mdh2	44.97	39.64	38.17	32.25	38.46	11	10	9	7	9	2	2	2	2	1	338	35.7
Q66HF1	NADH-ubiquinone oxidoreductase 75 kDa subunit, mitochondrial	Ndufs1	33.98	29.16	32.74	31.91	20.77	17	15	15	15	9	1	0	0	1	0	727	79.4
P10860	Glutamate dehydrogenase 1, mitochondrial	Glud1	25.27	31.18	32.44	23.12	27.96	10	11	11	8	10	0	0	1	1	0	558	61.4

C: untreated control; M: monomers; O: oligomers; 100: 100 nM; 500: 500 nM

One of the most common modifications was found to be oxidised methionine. Beside cysteine, methionine is another sulphur-containing AA implicated in the antioxidant protection of proteins in order to preserve their structure and function^20,21^. Although oxidation of methionine can be reversed, this PTM may act as a regulatory switch, and can inhibit the phosphorylation of adjacent amino acids sites, such as serine, threonine, or tyrosine, thereby altering protein function. However, the function of oxidation of the sites in ATP synthase highlighted with this data, is not yet known.

### Oligomer-induced toxicity is dependent on PTP opening

To investigate whether oligomer-induced PTP opening was responsible for oligomer-induced toxicity, monomeric (500 nM) or oligomeric α-synuclein was applied to cells overnight and cell death was quantified by assessing the ratio of propidium iodide (PI) and Hoechst positive cells. Oligomers (500 nM monomer, ~5 nM oligomers) alone induced a significant increase in cell death compared to buffer control (*p* < 0.001; Fig. 4j, k). Co-incubation with a PTP inhibitor (CsA) was able to reduce this cell death significantly (*p* < 0.05). Interestingly, co-incubation with a mitochondrial-located antioxidant (MitoQ) was also effective in preventing oligomers induced cell death.

### SNCA triplication iPSCs exhibit early PTP opening

In familial PD, there is a correlation between the number of SNCA alleles and severity of pathology^22^. To recapitulate the SNCA gene dosage effect in human systems, we utilised two models: (1) transgenic Shef4 human embryonic stem (hES) cells engineered to overexpress (OE) two different levels of SNCA, on an isogenic background (Supplementary Fig. 4A), and (2) inducible pluripotent stem cells (iPSCs) from a PD patient with a triplication in the *SNCA* locus, a control unaffected first-degree relative^23^ and an isogenic control (Supplementary Fig. 5A, B). iPSC-derived cortical neurons from three disease, three control independent clones, and one isogenic control clone were generated using standard protocols, and experiments were performed on a minimum of three independent inductions. These protocols generated cultures that were 90% neuronal (as demonstrated by Tuj1 positive staining), and 70% of the neurons demonstrated glutamate-induced calcium responses (Supplementary Fig. 5A, B).

**Fig. 5:**
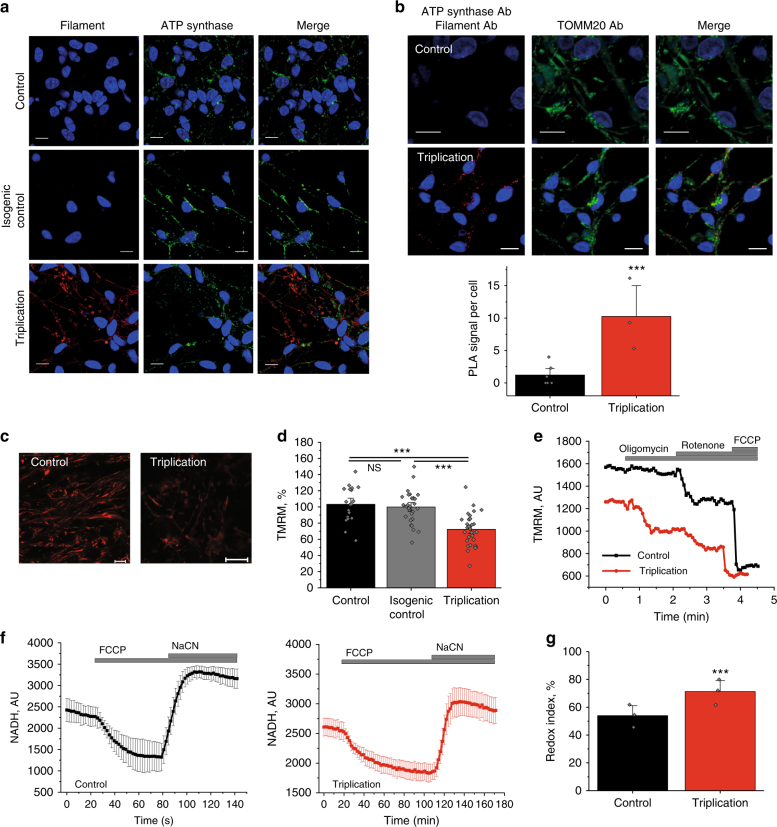
Impaired mitochondrial function in SNCA triplication iPS derived neurons. **a** Representative ICC images of control, isogenic control, and triplication cells probed for filament α-synuclein. The nucleus can be seen in blue (DAPI). **b** Representative PLA images and quantification of control and triplication iPS derived neurons showing a close proximity between filament α-synuclein and ATP synthase with the nucleus in blue (DAPI). TOMM20 was probed for to validate the mitochondrial localisation of the PLA signal. *N* = 3 experiments; control *n* = 155 cells, and triplication *n* = 115 cells. **c** Representative images of control and triplication iPS derived neurons loaded with TMRM and **d** ΔΨm quantification. *N* = 3 experiments, *n* ≥ 32 cells. **e** Representative traces of TMRM fluorescence in control and SNCA triplication neurons after addition of oligomycin (2 μg/ml), rotenone (1 μM), and FCCP (1 μM). **f** Representative trace of NADH in control and triplication iPS derived neurons. FCCP is applied to maximise respiration and therefore minimise the NADH pool and NaCN is added to block the mitochondrial respiration and therefore maximise the NADH pool. **g** Quantification of the redox index in control (*N* = 3 experiments, *n* = 112 cells) and triplication iPS derived neurons (*N* = 3 experiments, *n* = 148 cells). Two-tailed Student’s *t*-test for **b**, **d**, **g**. **b** Scatter points on scatter column represent number of puncta per cell. **d**, **g** Scatter points represent individual cells/areas. Data represented as mean ± SEM. *p < 0.05; **p < 0.01; ***p < 0.001

Immunocytochemistry using the conformation-specific filament α-synuclein antibody demonstrated a higher signal in the triplication neurons (Fig. 5a), suggesting higher levels of endogenous aggregated α-synuclein in triplication neurons compared to control and isogenic control. PLA showed a significant increase in the number of fluorescent puncta in the SNCA triplication neurons showing that endogenous aggregated α-synuclein in the iPSC-derived neuronal model interacts with the ATP synthase (*p* < 0.001; Fig. 5b). Puncta could also be observed in control iPSC neurons potentially reflecting the existence of low levels of physiological conformers of α-synuclein in control neurons. The mitochondrial protein, TOMM20, was used to validate the mitochondrial localisation of the PLA signal.

∆ψm was assessed in hES and iPSC-derived neurons using TMRM. At days 70–90 post-differentiation, triplication neurons demonstrated reduced ΔΨm compared to control and isogenic control cells (Fig. 5c, d; hESC α-syn OE, Supplementary Fig. 4B). Further, depolarisation upon application of oligomycin (complex V inhibitor) revealed that the ΔΨm in triplication neurons is maintained by the ATP synthase working in the reverse mode (ATPase; Fig. 5e; hESC α-syn OE, Supplementary Fig. 4C). Interestingly, the NADH redox index, which was assessed in % by maximal activation of NADH mitochondrial consumption by FCCP (0%), and maximal inhibition of NADH consumption by mitochondrial ETC inhibitor, sodium cyanide (100%) in triplication neurons was significantly higher compared to control (*p* <0 .01; Fig. 5f, g) suggesting that the resting level of NADH was elevated due to impaired consumption of NADH by complex I (hESC α-syn OE; Supplementary Fig. 4D, E). Importantly, isogenic control cells had a comparable NADH redox index to control neurons (Supplementary Fig. 5C).

**Fig. 6:**
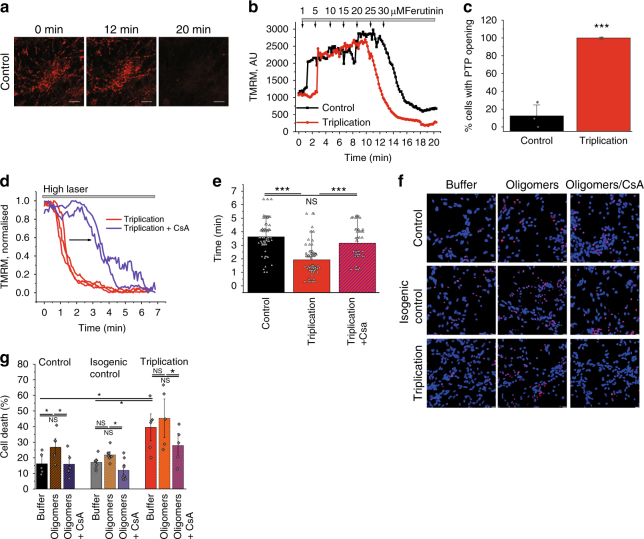
Lowered PTP opening threshold and increased cell death in SNCA triplication iPS derived neurons. **a** Representative images of mitochondria labelled with TMRM before ferutinin was added (0 min), immediately before mPTP opening (12 min), and after mPTP opening (20 min). **b** Representative traces of PTP opening in control and triplications in response to ferutinin. **c** Proportion of cells (%) that fully opened PTP in response to 25 μM ferutinin. *N* = 3 experiments, control *n* = 125 cells, and triplication *n* = 82 cells. **d** Representative traces of PTP opening in SNCA triplication neurons ± CsA in response to high laser. **e** Quantification of the time until laser-induced PTP opening in control (*N* = 3 experiments; *n* = 77 areas), SNCA triplication neurons (*N* = 3 experiments; *n* = 82 areas), and SNCA triplication neurons + CsA (*N* = 3 experiments; *n* = 49 areas). **f** Representative images and **g** quantification of cell death in control, isogenic control, and triplication neurons challenged with control buffer or oligomers ± CsA. *N* = 3 experiments, *n* ≥ 763 cells. **g** Scatter points represent individual fields of view. Two-tailed Student’s *t*-test for **c**. One-way ANOVA with Bonferroni correction (**e**, **g**). Scatter points represent sampled fields of view. Data represented as mean ± SEM. Scale bar = 10 μm. *p < 0.05; **p < 0.01; ***p < 0.001

The mitochondrial calcium capacity determines the threshold at which PTP opening occurs. Whole neurons were loaded with TMRM (Fig. 6a) and challenged with increasing concentrations of the calcium ionophore ferutinin in a stepwise fashion (Fig. 6b). The electrogenic calcium ionophore ferutinin is able to induce a rise in mitochondrial calcium^24–26^, and here the concentration of ferutinin required to induce PTP opening (confirmed by the rapid loss of TMRM signal; Fig. 6b) was determined. PTP opening occurred at significantly lower concentrations of ferutinin in triplication neurons when compared to control (*p* < 0.001; Fig. 6c). Thus, the lower calcium-induced PTP opening threshold in the triplication neurons suggests that the aggregated forms of α-synuclein within the cell lead to premature PTP opening. The PTP opening threshold in triplication neurons was further tested in response to laser-induced ROS. Administering a high laser power induced earlier PTP opening in triplication lines compared to control cells which could be delayed by preincubation of the neurons with CsA (*p* < 0.001; Fig. 6d, e; hESC α-syn OE, Supplementary Fig. 4F-H).

Vulnerability to cell death of the triplication neurons was assessed in the presence or absence of oligomers (Fig. 6f). Basal cell death was increased in triplication neurons compared to control and isogenic control neurons (Fig. 6g). Incubation with 500 nM oligomeric (~5 nM oligomers) α-synuclein significantly increased the basal death in controls (*p* < 0.05; Fig. 6g). Co-application of the PTP inhibitor CsA prevented the oligomer-induced toxicity and restored cell death levels back to basal.

In human SNCA triplication and ES α-synuclein OE neurons, high levels of endogenously aggregated α-synuclein lead to lower ΔΨm, impaired mitochondrial respiration and a lower threshold for PTP opening.

## Discussion

Studies in cell lines and transgenic mice have previously observed mitochondrial pathology associated with synucleinopathy^27–32^. However, the nature of the toxic α-synuclein aggregate, and the mechanism by which it induces mitochondrial pathology remains unresolved. Applying single-molecule methods (to select aggregates by their structure and size) to isolated mitochondria, primary rodent neurons, and human iPSC-derived neurons permitted a tractable mechanism of α-synuclein induced toxicity to be resolved. Our study identifies that α-synuclein oligomers induce mitochondrial dysfunction, ultimately leading to neuronal death by activating the mitochondrial PTP. The mitochondrial PTP may therefore represent a convergence between protein aggregation and neuronal mitochondrial dysfunction in PD.

Super-resolution microscopy, and proximity ligation assays, confirmed that α-synuclein aggregates can localise to the mitochondrial membrane of rodent and human neurons, where they come into close proximity with a number of key mitochondrial proteins. In accordance with previous reports^12^, we show that α-synuclein oligomers interact with, and impair complex 1 respiration, leading to slow depolarisation of the mitochondrial membrane potential. Of major importance in this study, we demonstrated that the soluble, β sheet-rich oligomers of α-synuclein directly induce PTP opening. PTP opening is an event in which an increase in permeability of the inner mitochondrial membrane occurs due to opening of a high conductance megachannel in the inner mitochondrial membrane called the PTP, leading to equilibration of ionic gradients and solutes, swelling of the matrix, cristae unfolding, rupture of the outer mitochondrial membrane and ultimately cell death^33–35^. We deployed a range of methods, including (1) fluorescent imaging of rapid mitochondrial depolarisation in whole cell preparations, (2) mechanical swelling assay of isolated mitochondria, and (3) electrophysiological detection of a megachannel in mitoplasts, which together confirmed that oligomeric but not monomeric α-synuclein, can induce PTP. The molecular identity of PTP remains controversial. In early studies, proposed candidates have included ANT, VDAC, phosphate carrier PiC, and misfolded proteins^36–40^. More recent evidence suggests that the ATP synthase components can be critically involved in the formation of PTP channel either through reorganisation of C-subunit ring or through dimerisation of ATP synthase complex, although an essential role of ATP synthase in PTP formation has been questioned^41–47^. In this study, we do not attempt to define the composition of the PTP, especially in light of the debate in the literature regarding this. However, our data suggests that the probability of PTP opening may be regulated by an interaction between monomeric and oligomeric α-synuclein with ATP synthase, and that oligomer-induced oxidation events increase the probability of PTP opening in disease. Understanding why the ‘pathological’ oligomer specifically opens the PTP, while the ‘physiological’ monomer is unable to do so, is fundamental to understanding how aggregation induces mitochondrial toxicity.

One major difference between monomer and oligomer lies in the enriched β-sheet, hydrophobic structure that enables the oligomer to interact with the inner mitochondrial membrane and form channels, which we have demonstrated occurs in isolated mitochondria preparations. The second major difference is the unique intrinsic property for oligomeric structures to generate ROS independently of cellular enzymatic systems, a property that has been recognised for other aggregates of amyloidogenic proteins^48^. Interestingly we have previously shown that, in its unfolded state, the monomeric form of α-synuclein interacts with ATP synthase. Here we utilise a conformation-specific antibody combined with PLA and super-resolution microscopy to demonstrate that, in the oligomeric state, α-synuclein still comes into close proximity with ATP synthase. Whilst both monomers^10^ and oligomers can interact with the ATP-synthase, they exert different effects on the ATP synthase function: monomers improve the efficiency of ATP synthesis^10^. In contrast, one major consequence of oligomers when applied to isolated mitochondria is to induce oxidative modifications, specifically to the ATP synthase β subunit, demonstrated by redox proteomics. The ATP synthase is known to be susceptible to oxidative/nitrosative stress^49^. PTMs of specific residues of ATP synthase induced by ROS can affect both the catalytic activities of ATP synthase and potentially induce its PTP-forming activity^31^. Redox-active oligomeric complexes are therefore able to oxidise their target proteins in the inner mitochondrial membrane, and additionally induce lipid peroxidation of the mitochondrial membrane. Notably, free radical scavenger/antioxidant in isolated mitochondrial preparations (using Trolox) and whole cells (MitoQ), prevents the oligomer-induced PTP opening, presumably through preventing oligomer-induced oxidation events. This supports the conclusion that the oligomer induces the PTP via inducing oxidation of target proteins and mitochondrial lipids, while the monomer is unable to so.

This mechanism may be relevant in the human brain during the course of PD. SNCA mutations, and in particular the SNCA triplication, lead to an early onset autosomal dominant form of PD with dementia, associated with widespread α-synuclein aggregation throughout the cortex and midbrain. Moreover, there is a correlation between the SNCA gene dosage (and α-synuclein expression level) and disease severity. We utilised human neurons expressing different levels of endogenous α-synuclein on an isogenic background, and control and iPSC-derived neurons bearing a SNCA triplication, to model the effect of increased expression and aggregation of α-synuclein in disease. Elevated endogenous expression of α-synuclein induces mitochondrial dysfunction, characterised by impaired respiration and mitochondrial membrane depolarisation. Notably endogenous α-synuclein aggregates in these models also interact with mitochondrial proteins, impair respiration, and induce early PTP opening and neuronal death. The neuronal death in these models can be prevented by the use of PTP inhibitors, or by preventing oligomer-induced oxidation events within the mitochondria.

**Fig. 7:**
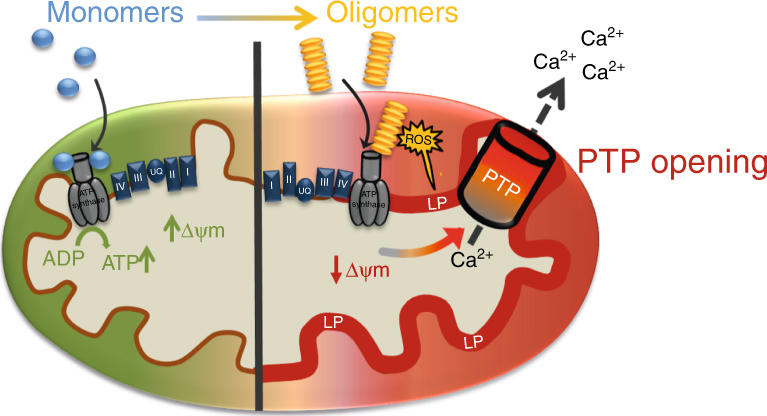
Schematic diagram of oligomeric α-synuclein effects on mitochondria. Monomeric α-synuclein interacts with ATP synthase and improves the efficiency of ATP synthesis. Oligomeric α-synuclein also interacts with ATP synthase but conversely, impairs respiration, and depolarises the mitochondria. Oligomers induce ROS production, leading to lipid peroxidation and oxidation of key mitochondrial proteins. Together, these oligomer-induced events open the mitochondrial permeability transition pore

Our data proposes the hypothesis that monomeric form of α-synuclein, under normal conditions, interacts with and enhances the efficiency of ATP synthase. During disease, aggregation of monomeric α-synuclein is triggered, and this generates a series of intermediate oligomers with increasing enrichment of β sheet structures. These structures are in close proximity to inner mitochondrial membrane proteins, and act to inhibit complex 1 function, and generate targeted oxidation events on interacting proteins, and lipids. Aggregates of α-synuclein generate a pool of bioenergetically compromised mitochondria under oxidative stress. Oligomer-induced oxidation events ultimately lead to opening of a megachannel, the mitochondrial permeability transition event (Fig. 7). Additionally, oligomers sensitise cells to known PTP triggers as they induce increased levels of ROS production and calcium dysregulation, lowering the threshold for PTP opening in response to other stimuli^8,9,50^. Aging is a major factor in PD, and although difficult to recapitulate in vitro, it is likely that age-related decline in bioenergetics, redox homeostasis, and mitochondrial calcium capacity, contribute to the aggregate induced pathology to accelerate pathogenesis. Mitochondrial PTP opening may therefore be a common pathway by which oligomeric α-synuclein induces cell death in human neurons. This work resolves how a change in the structure of a protein transitioning to its oligomeric state, alters its functional consequence. Such insights are fundamental to understanding how the process of protein aggregation generates toxic oligomeric species that may be directly pathogenic in neurodegenerative diseases.

## Methods

### Animals

Sprague–Dawley rat pups 1–3 days postpartum (University College London breeding colony) of either sex were used for neuronal co-cultures and experimental procedures were performed in full compliance with the United Kingdom Animal (Scientific Procedures) Act of 1986.

### WT rat neuronal co-cultures

Cultures of midbrain neurons and glial cells were prepared from postnatal Sprague–Dawley pups (day 1–3; UCL breeding colony). Midbrains were placed in ice-cold HBSS (Ca^2+^, Mg^2+^-free; Invitrogen). The tissue was minced, trypsinized (0.1% for 10 min at 37 °C), triturated and plated on poly-D-lysine-coated coverslips. The cells were cultured in Neurobasal medium (Invitrogen) supplemented with B-27 (Invitrogen), 2 mM L-Glutamine and penicillin (50 I.U./ml)/streptomycin (50 μg/ml). The cultures were maintained at 37 °C (5% CO_2_) and the media changed twice a week. Cells were used at 12–15 days in vitro. Neurons were easily distinguishable from glia as they appeared brighter with smooth rounded somata with distinct processes. Neurons lay just above the focal plane of the glial layer.

### iPSC-derived neurons

iPSC control lines SFC840-03-01, SFC840-03-03, and SNCA triplication lines SFC831-03-01, SFC831-03-03, and SFC831-03-05 were derived and characterised at the University of Oxford, James Martin Stem Cell Facility, and control iPSC line SBAD04-01 was derived and characterised at the University of Newcastle from Lonza fibroblasts CC-2511, Lot 264781, by the StemBANCC consortium^51,52^. iPSC lines AST3 and AST8 were derived from a patient with a SNCA triplication, NAS6 and NAS7 were derived from an unaffected first-degree relative^23^. iPSCs were cultured on matrigel in Essential 8 medium (ThermoFisher) and passaged using collagenase. Differentiation of cells into neurons was induced by dual SMAD inhibition using SB431542 (10 μM, Abcam) and LDN (100 nM Abcam) in knockout DMEM (ThermoFisher), followed by maturation in N2B27 medium (ThermoFisher) supplemented with BDNF and GDNF (20 ng/ml and 10 ng/ml, Peprotech)^53^. Cells were passaged using accutase after 11 days, and seeded onto plates coated with poly-l-ornithine, laminin and fibronectin (Sigma). Cells were passaged approximately three more times before being used at days 70–90 after the start of differentiation. iPSC-derived cortical neurons from three patients (SNCA ×3) clones and three control clones were generated using standard protocols, and all experiments were performed on a minimum of three independent inductions. Experiments were repeated using the SNCA ×3 clones, and an isogenic clone generated from the same patient. Briefly, the isogenic cell line was generated from a SNCA ×3 iPSC clone by CRISPR/Cas9 double nickase gene editing to knockout 2 SNCA alleles, reducing the allele dosage from four (in the triplication cells) to two (normal), as confirmed by DNA sequencing. This method retains the rest of the triplication locus intact, and therefore provides the ideal control for the effects of SNCA triplication alone.

### hES-derived neurons

The hESC line was kindly provided by Dr. David Hay (University of Edinburgh), upon MRC Steering Committee approval (ref. no. SCSC11-60). The line was established at the Centre for Stem Cell Biology (University of Sheffield) under a license from the Human Fertilisation and Embryology Authority, and has been validated to show the standard hESC characteristics including a normal karyotype^33^. Neural induction by dissociation of hES cells into single cells with Accutase (Gibco, Cat. no. A11105-01) and plated on a Matrigel-coated 6-well plate in mTeSR1 medium. Cells were fed daily until they reached 90% confluency or above. Neural induction started at day 0, when mTeSR1 was replaced with hESC medium lacking FGF2, supplemented with 10 μM SB431542 (Tocris) and 100 nM LDN-193189 (Stemgent). Cells were fed daily with this medium until day 4. From day 5 to day 11, SB431542 was withdrawn and cells were fed every other day with a mixture of hESC medium and N2B27, which was gradually added into culture medium from 25%, 50%, 75%, and 100% at day 5, day 7, day 9, and day 11, respectively^53^. To generate cortical neurons, human pluripotent stem cells were processed based on a protocol described above for the iPSC-derived neurons^54^. pCAG-SNCA-IRES-Venus or the control pCAG-IV were transfected into hES cells followed by antibiotic selection to allow the generation of clones with stable expression of SNCA. Clones exhibiting normal morphology, growth and differentiation behaviour were selected and characterised for SNCA expression, and two clones with near normal levels of SNCA expression (here designated control) and high levels of SNCA expression (designated as hES OE syn) were utilised for further studies.

### Aggregation of α-synuclein

For the aggregation reactions, a 70 μM solution of wild-type α-synuclein in 25 mM Tris buffer with 100 mM NaCl (pH 7.4; 0.01% NaN_3_ was added to prevent bacterial growth during aggregation) was incubated at 37 °C with constant agitation at 200 rpm (New Brunswick Scientific Innova 43), during which time aliquots were taken. All aggregations were conducted in LoBind microcentrifuge tubes (Eppendorf) to limit surface adsorption.

### Preparation of ThT solution

ThT stock solutions were prepared by diluting ThT (Sigma-Aldrich, T3516) into neat ethanol (Sigma-Aldrich, 459836) to give a final concentration of ∼1 M. Following this, a dilution into PBS was performed to give a ∼50 μM stock solution of ThT. The ThT solution was freshly filtered each day (0.02 μm syringe filter, Whatman, 6809–2101), and the exact concentration was determined from the absorbance at 412 nm using an extinction coefficient of 36 000 M^−1^ cm^−1^. The stock solution was stored in the dark at 4 °C, and was only used for a maximum of 2 weeks after preparation.

### Preparation of slides for SAVE imaging

Borosilicate glass coverslips (20 × 20 mm, VWR, 631-0122) were cleaned using an argon plasma cleaner (PDC-002, Harrick Plasma) for at least 1 h. Frame-seal slide chambers (9 × 9 mm^2^, Biorad, Hercules, CA, SLF-0601) were attached to the glass, and 50 μL of poly-L-lysine (70 000–150 000 molecular weight, Sigma-Aldrich, P4707) was added to the well and incubated for at least 30 min, before being washed with PBS buffer. Prior to imaging, the coverslides were first checked for fluorescent impurities in 5 μM ThT.

### SAVE imaging

Imaging was performed using a homebuilt total internal reflection fluorescence microscope^11^. This imaging mode restricts detectable fluorescence signal to within 200 nm from the sample slide. For imaging of WT α-synuclein in the presence of 5 μM ThT, the output from laser operating at 405 nm (Oxxius LaserBoxx, LBX-405-100-CIR-PP) was aligned and directed parallel to the optical axis at the edge of a 1.49 NA TIRF objective (APON60XO TIRF, Olympus, N2709400), mounted on an inverted Nikon Eclipse TiE microscope. Fluorescence was collected by the same objective and was separated from the returning TIR beam by a dichroic (Di01-R405/488/561/635, Semrock), and passed through an emission filter (BLP01-488R). The images were recorded on an EMCCD camera (Evolve 512, Photometrics) operating in frame transfer mode (EMGain of 11.5 e–/ADU and 250 ADU/photon). Each pixel was 160 nm in length. For each data set, 3 × 3 image grids were measured in three different regions of the cover slide. The distance between the nine images measured in each grid was set to 350 μm, and was automated (bean-shell script, micromanager) to prevent user bias. Images were recorded at 50 frames s^−1^ for 100 frames with 405 nm illumination (150–200 W/c^2^).

### Data analysis of SAVE images

Data analysis was performed using a custom-written script in Igor Pro (Wavemetrics). For each image, the stacks were first averaged over 100 frames, and the background subtracted. Fluorescent species were detected by applying a threshold of five standard deviations above the mean image intensity, and were subsequently fit to two-dimensional elliptic Gaussian distributions. The integrated intensities of the fits and the maximum widths were determined and presented in histograms (Fig. 1b).

### Single channel patch-clamp recordings

Single channel patch-clamp experiments were performed using mitoplasts prepared from isolated mouse brain mitochondria. For mitochondrial isolation, brain tissue was homogenised in 3 ml of isolation buffer containing 70 mM sucrose, 230 mM mannitol, 2 mM EGTA, 5 mM Hepes-KOH (pH 7.4), and centrifuged at 800×*g* for 15 min. Supernatant was centrifuged at 10 000×*g* for 15 min. Pellet containing isolated mitochondria was resuspended in buffer containing 70 mM sucrose, 230 mM mannitol, and 5 mM Hepes-KOH (pH 7.4). Mitoplasts were prepared by following methods: (1) passive swelling in buffer conation 150 mM KCl, 5 mM Hepes-KOH (pH 7.4), yielding mitoplasts without PTP; (2) Ca^2+^ induced swelling in buffer containing 1 mM CaCl_2_, 1 mM KH_2_PO_4_, 150 mM KCl, 5 mM Hepes-KOH, pH 7.4 in the presence of 5 mM succinate and 1 µM rotenone, yielding mitoplasts containing PTP. Patch clamp recordings were done using borosilicate pipettes (20 MΩ) in symmetrical solution containing 150 mM KCl, 5 mM Hepes-KOH (pH 7.4) using eONE amplifier and acquisition software. Data were analysed using Clampfit software (Molecular Devices).

### Isolation of mitochondria

Mitochondria were isolated from the brain of Sprague–Dawley rats (200–250 g) where the animal execution was performed according to the protocol approved by the Animal Care Committee of Dalhousie University. Brain was homogenised with a Potter–Elvehjem homogenizer in the mitochondrial isolation buffer (300 mM sucrose, 2 mM EDTA, 5 mM Tris–HCl, and 0.5 mg/ml bovine serum albumin, pH 7.4). Nuclei and unbroken cells were spun at 600×*g* for 10 min, supernatant was collected and spun at 6000×*g* for 20 min. The resulting pellet was re-suspended in 30 ml of the isolation buffer without EDTA and BSA and spun at 7500×*g* for 20 min. The resulting pellet (mitochondria) was re-suspended in 0.5 ml of the isolation buffer without EDTA and BSA and put on the ice-bath.

### Mitochondrial swelling assay

PTP was induced by the addition of small portions of calcium to energised mitochondria. PTP opening was measured by classical method of mitochondrial swelling^55^, which was detected by an in-house-modified Quantamaster-4 Spectrofluorimeter (PTI, Birmingham, NJ) at 540 nm wavelength. Experiments were performed at room temperature with continuous mixing. Complete mitochondrial swelling in some experiments was achieved by the addition of 5 μM of alamethicin (ala). Mitochondria were added in the final concentration 0.5 mg/ml, evaluated in terms of protein concentration, in recording buffer (Tris–HCl 5 mM; Sucrose 70 mM; Manitol 210 mM; glutamate 5 mM; malate 1 mM; KH_2_PO_4_ 0.2 mM, pH = 7.4).

### Live cell imaging

For TMRM experiments, cells were incubated with 25 nM TMRM in a HEPES-buffered imaging solution (Invitrogen) for 40 min at room temperature. Measurements were obtained by using a Zeiss 710 VIS CLSM equipped with a META detection system and a 40× oil-immersion objective while keeping 25 nM TMRM in the imaging solution. TMRM was excited using the 560 nm laser line and fluorescence was measured >580 nm. Z-stack images were obtained by confocal microscopy and the basal ΔΨm was measured using Zen software (Zeiss). Assessments of the mitochondrial membrane potential maintenance through application of oligomycin, rotenone, and the uncoupler FCCP were performed through recordings from a single focal plane. TMRM was used in the redistribution mode to assess the ΔΨm, meaning that a reduction in TMRM fluorescence represents mitochondrial depolarisation.

Alternatively, cells were loaded with Rh123 (2 μM; Invitrogen) for 15 min and washed with HBSS prior to the experiment. These loading conditions are nontoxic and provide ΔΨm measurements through the dequench mode of mitochondrial fluorescence where an increase in the cytosolic Rh123 fluorescence (measured from whole cells) redistributed from the mitochondria, reflects mitochondrial depolarisation. Excitation light was provided by a xenon arc lamp with the beam passing sequentially through 10 nm band-pass filters centred 490 nm housed in computer-controlled filter wheel (Cairn Research, UK). Emitted fluorescence light was reflected through a 515 nm long-pass filter to a cooled CCD camera (Hamamatsu, Orca ER) and digitised to 12 bit resolution (Digital Pixel Ltd., UK). Imaging data were collected and analysed using Andor iQ software (Belfast, UK). The signals were normalised between resting level (set to 0%) and a maximal signal generated in response to the uncoupler FCCP (1 μM; set to 100%). In some experiments, 10 µM cyclosporin H was used to block multidrug-resistant pump. No difference in results ± cyclosporine H has been observed in these cell models.

NADH autofluorescence was measured using an epifluorescence inverted microscope equipped with a 40× oil-fluorite objective. Excitation at a wavelength of 360 nm was provided by a xenon arc lamp, with the beam passing through a monochromator (Cairn Research). Emitted light was reflected through a 455 nm long-pass filter to a cooled CCD camera (Retiga; QImaging) and digitised to 12 bit resolution. Imaging data were collected and analysed using software from Andor.

Superoxide production was measured by using dihydroethidium (HEt; 2 μM, Invitrogen) and HBSS. Fluorescent images were acquired using an epifluorescence inverted microscope equipped with a 20× fluorite objective (frame interval of 10 s). The ratio of oxidised and reduced forms of the dye was measured as follows: excitation was set to 530 nm and emission was recorded above 560 nm to allow quantification of the oxidised form (ethidium), whereas excitation at 380 nm and emission from 405 to 470 was used to record the reduced form (hydroethidium). Data were analysed using software from Andor IQ (Belfast, UK).

Lipid peroxidation was measured using confocal microscopy (Zeiss 710 LSM with an integrated META detection system). The rate of lipid peroxidation was measured using C11-BODIPY 581/591 (2 μM; Molecular Probes) which was excited by the 488 and 543 nm laser line and fluorescence measured using a band-pass filter from 505 to 550 nm and 560 nm long-pass filter (40× oil-objective). Illumination intensity was kept to a minimum (0.1–0.2% of laser output) to prevent phototoxicity and the pinhole was set to give an optical slice of ∼2 μm. Addition of a bright-field image allowed separation between neurons and glia that are visibly different and are situated on different focal planes.

### ICC and PLA

Cells were fixed in 4% paraformaldehyde and permeabilized with 0.2% Triton-100. 5% BSA was used as to block non-specific binding before cells were incubated with primary antibodies overnight at 4 °C (1:500 R&D Systems TuJ1 antibody MAB1195; 1:500 BD α-synuclein antibody 610787; 1:100 Abcam fibrillar synuclein antibody ab209538 and 1:100 ATP synthase subunit-α antibody ab14748; BD MAP2 Alexa488 560399; 1:100 Abcam MTCO1 ab14705; 1:200 Abcam Histone H3 ab1220; 1:100 Abcam TOMM20 Alexa488 ab205486). For ICC, cells were probed with 1:500 Alexa-Fluor 488 (Thermo-Fisher) and 1:500 Alexa-Fluor 594 (Thermo-Fisher) for 1 h at RT. The PLA was carried out using the Duolink in situ red kit (mouse/rabbit; Sigma; DUO92101) according to the manufacturer’s instructions. The mounting media contained DAPI to allow for nuclear staining.

### Redox proteomics

For redox proteomics, rat brain mitochondria were isolated as described above. The mitochondrial sample was divided and treated with either buffer, monomers or oligomers for 15 min. Mitochondrial protein samples were either directly separated on a SDS-PAGE using LDS non-reducing sample buffer (Invitrogen 84788) or a co-IP was performed to pull-down ATP synthase (ab109715) and the eluent was separated on a SDS-PAGE using LDS non-reducing sample buffer. The whole sample lines were divided into eight equal parts, excised and destained overnight. Digestion of proteins was carried out similarly as described elsewhere^56^. Samples were washed for 2 h with 5% acetic acid/methanol (50/50, v/v), followed by dehydration with neat acetonitrile in a vacuum centrifuge (Eppendorf, Hamburg, Germany) for 5 min. Protein samples were then, reduced with 10 mM dithiothreitol in 100 mM ammonium bicarbonate, alkylated with 100 mM iodoacetamide in 100 mM ammonium bicarbonate, and digested overnight at 37 °C using 0.6 μg of trypsin gold (Promega) in 50 mM ammonium bicarbonate. Peptides were extracted with 45% water/50% acetonitrile/5% FA and the volume was reduced to 20 µl using the vacuum centrifuge^57^.

The liquid chromatography-tandem mass spectrometry (LC-MS/MS) analysis was performed using shotgun separation of peptide mixtures on a 25 cm reversed-phase C18 column (75 µm, 2 µm Acclaim RSLC C18, Thermo Scientific, Waltham, USA) by Easy n-LC II (Thermo Scientific, Waltham, USA) coupled to an Orbitrap Fusion tribrid mass spectrometer (Thermo Scientific, Waltham, USA). The elution was carried out over 108 min step gradient at a constant flow rate of 300 nL/min. Elution started with 5% acetonitrile (with 0.1% FA), ramping to 37% acetonitrile (with 0.1% FA) from 5 to 95 min, then ramping to 80% acetonitrile (with 0.1% FA) at 99 min, hold at 80% acetonitrile (with 0.1% FA) for 2 min and back to 5% acetonitrile (with 0.1% FA) at 108 min.

The Orbitrap Fusion Tribrid analysis was set to data-dependent acquisition using FTMS master scan preview mode, mass range 400–1600 *m*/*z* at 120 000 resolution, for triggering MS/MS events. FTMS data were recorded with a max injection time of 100 ms, automated gain control (AGC) at 200 000, positive ion lock mass of 445.12003 *m*/*z* and orbitrap as a detector. Monoisotopic precursor selection filter and dynamic exclusion with 30 s duration was used. Tandem MS was performed in top-speed mode (3 s cycle time) by quadrupole isolation using HCD fragmentation with the 30% of normalised collision energy, max injection time of 70 ms, AGC at 10 000 and ion trap as detector.

MS data were analysed with SEQUEST HT search engine in Proteome Discoverer 1.4 (Thermo Scientific, San Jose, CA, USA) against Uniprot Rattus norvegicus database (downloaded on 12th October 2017). The search parameters for HCD-ITMS fragment ions used were as follows: 10 ppm precursor mass tolerances, 0.6 Da fragment mass tolerance, maximum of two missed cleavages were allowed for tryptic peptides, min/max precursor mass was set to 350/5000 Da, the peptide false discovery rate calculated by a target–decoy approach was set to 0.01. Additionally, 12 different types of variable modifications of peptides were searched. The modifications included were as follows: carbamidomethylation of cysteine, C-terminal oxidation and terminal independent carbonylation (+13.97926 Da), mono-oxidation (+15.99491 Da), di-oxidation (+31.98982 Da), tri-oxidation (+47.985 Da), oxidation of His to Asn (−23.0159 Da) or Asp (−22.032 Da) and oxidation of lysine to aminoadipic acid (+14.9632 Da) (Supplementary Table 1). Peptides with medium (*p* < 0.05) and high confidence (*p* < 0.01) were used for initial filtering of data for comparative redox proteomics analysis.

To quantify the ratio of peptides with oxidised methionine vs non-oxidised ([Met(O)]/[Met]) according to Suzuki et al.^57^, peptide ion intensities were extracted from the MS/MS files of representative samples using Precursor Ions Area Detector node in Proteome Discoverer. The degree of oxidation was measured by comparing the integrated peak areas of the [Met(O)]/[Met] peptides. The mass spectrometry proteomics data have been deposited to the ProteomeXchange Consortium via the PRIDE^58^ partner repository with the dataset identifier PXD009416.

### Cell toxicity experiment

Cells were incubated with PI (20 μM) and Hoechst 33342 (4.5 µM; Molecular Probes, Eugene, OR). Viable cells exclude the red fluorescent PI whereas Hoechst stains chromatin blue in all cells thus allowing dead cells to be quantified.

### Super-resolution

Two colour super-resolution was achieved using DNA PAINT^15^. Prior to imaging, the cells were fixed in 4% paraformaldehyde and permeabilized with 0.2% Triton-100. 10% Goat Serum (Sigma Aldrich) was used to block non-specific binding before cells were incubated with primary antibodies overnight at 4 °C (Abcam; 1:100 fibrillar synuclein antibody ab209538; 1:100 ATP synthase subunit-α antibody ab14748). The primary antibodies were probed using biotinylated secondary antibodies bound to DNA oligonucleotides via a biotin–streptavidin sandwich, which were prepared according to Jungmann et al.[15]. Briefly, 5 µl of biotinylated DNA docking strand (100 µM) (ATDBio LTD., Southampton, UK), 5 µl streptavidin (5 mg/ml) (Sigma-Aldrich, S4762), and 90 µl PBS were incubated at RT whilst gently shaking for 30 min. 1 µl of biotinylated secondary antibody (1 mg/ml) was then added to the solution and incubated for a further 30 min. Filter columns (Amincon, 100 kDa) were used to purify the pre-assembled conjugates from the unreacted streptavidin–DNA. The sequences of the DNA strands attached to the antibodies (docking strands) and to the fluorophores (imaging strands) are given below. DNA-PAINT utilises a fluorophore labelled imaging DNA strand that transiently binds to a complementary docking strand conjugated to the secondary antibody that binds the primary antibody, allowing it to be imaged at an equivalent high precision >20 nm. The cells were incubated with both secondary antibodies at a concentration of 10 µg/ml for 1 h. The imaging strand was added to the cells at an imaging concentration of 1 nM. Controls utilising untreated cells, primary antibodies alone and secondary antibodies alone were performed in parallel.

Docking strand for secondary antibody to fibrillar antibody: Biotin-TTATACATCTA

Imaging strand for secondary antibody to fibrillar antibody: CTAGATGTAT-ATTO655

Docking strand for secondary antibody to ATP Synthase antibody: Biotin–TTATCTACATA

Imaging strand for secondary antibody to ATP Synthase antibody: TATGTAGATC-CY3B

For the TOMM20 and complex-1 imaging (Supplementary Fig. 3), the same sequences were used, only the biotin was exchanged for a thiol and directly conjugated to the secondary antibody using a maleimide-PEG2-succinimidyl ester^59^. Equivalent imaging with ATP5A is also shown in Supplementary Fig. 3. The association quotients, *Q*, were calculated for each of these images generated.

Images were recorded on a custom-built instrument consisting of an Nikon TiE inverted microscope with an infinity-corrected oil-immersion objective using TIRF illumination (Olympus UPlanApo TIRF, ×60, 1.49 NA) and detected on an EMCCD camera (Photometrics Evolve, EVO-512-M-FW- 16-AC-110). Images were acquired at 50 ms per frame operating in frame transfer mode with an election multiplication gain of 250 fold. We used a dichroic mirror (Semrock, Di01-R405/488/561/635), and emission filters for Cy3B (Semrock, LP02-568RS-25 and FF01-587/35-25) and Alexa-Fluor 647 (Semrock BLP01-635R-25). Images were acquired through excitation at 561 and 641 nm (∼4 kW/cm^2^) for 8000 frames. The diffraction limited images were generated by taking the standard deviation of the intensity over the frames of the image stack.

Super-resolution images were analysed using the GDSC Single Molecule Light Microscopy plugin Peak Fit in ImageJ (Alex Herbert, University of Sussex, UK) by fitting to a 2D Gaussian function using the Least Squared Errors method. A noise threshold of 20 was used, with a precision cut-off of 50 nm. To remove spurious localisations, cluster analysis of the super-resolution images was performed using DBSCAN compiled in the python^60^. A distance threshold of 1 pixel (135 nm) was used, and clusters were defined as those with more than 20 localisations; all other localisations were discarded. The resolution was determined by plotting a Fourier ring correlation curve (GDSC SMLM package)^61^ for each image and determining the spatial frequency at which the curve drops below 1/7^62^.

To determine the percentage co-localisation, α-synuclein localisations were defined as being coincident with the mitochondrial marker if their positions plus their fitting precisions were within mitochondrial marker localisations plus their fitting precisions. The number of chance coincident events was estimated by performing the same analysis with simulated ATP synthase images having the same densities of localisations.

The association quotient (*Q*) was calculated according to the following equation^63^:

1 $$Q = \frac{{C - E}}{{A - E}}$$

where *Q* is the association quotient, *C* is the number of coincident events in both filament and mitochondrial images, *E* is the estimated number of chance co-localisations, and *A* is the total filament localisations.

### Statistical analysis

Origin 9 software was employed for the statistical analysis and exponential curve fitting. Biological replicates are termed ‘*N*’; number of cells analysed is termed ‘*n*’. Data was assessed for normality using the Shapiro–Wilk test. Experimental data are shown as means ± SEM, with superimposed scatter points to demonstrate variance within datasets. Statistical analysis was performed using unpaired two-tailed Student’s *t* test to analyse differences between two groups, or a one-way ANOVA with a Bonferroni correction to analyse differences among three or more groups. Differences were considered to be significantly different if *p* < 0.05.

### Data availability

Data supporting the findings of this manuscript are available from the corresponding authors upon reasonable request. Proteomic data is available via ProteomeXchange with identifier PXD009416.

## Electronic supplementary material

Supplementary Information
