# Supplementary material for: α-synuclein oligomers interact with ATP synthase and open the permeability transition pore in Parkinson’s disease
Source: Nat Commun. 2018 Jun 12;9:2293. doi: 10.1038/s41467-018-04422-2 (PMC5997668; doi:10.1038/s41467-018-04422-2)
Supplement: Supplementary file 1 — Supplementary Information [file 41467_2018_4422_MOESM1_ESM.pdf]

α-synuclein oligomers interact with ATP synthase and open the permeability transition pore in Parkinson's disease

Ludtmann MHR et al

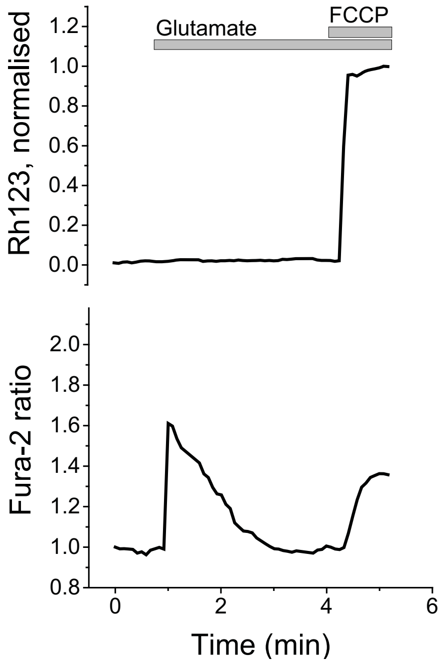

Supplementary figure1

Representative traces of Rh123 and Fura-2 of WT neurons exposed to glutamate.

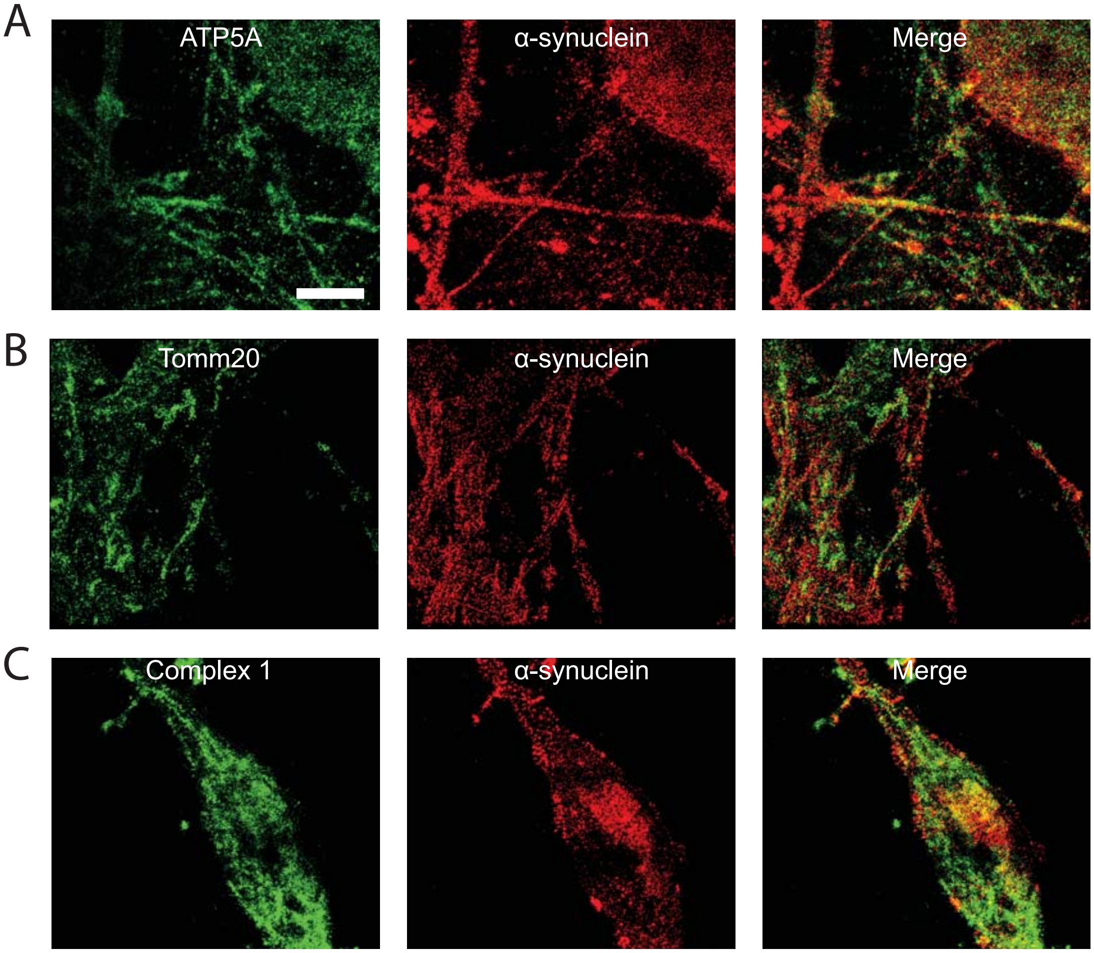

Supplementary figure 2
Super-resolved images of oligomer treated cells probed for (A) filament and ATP synthase, (B) TOMM20 and filament or (C) Complex 1 and filament. Scale bar = 5µm.

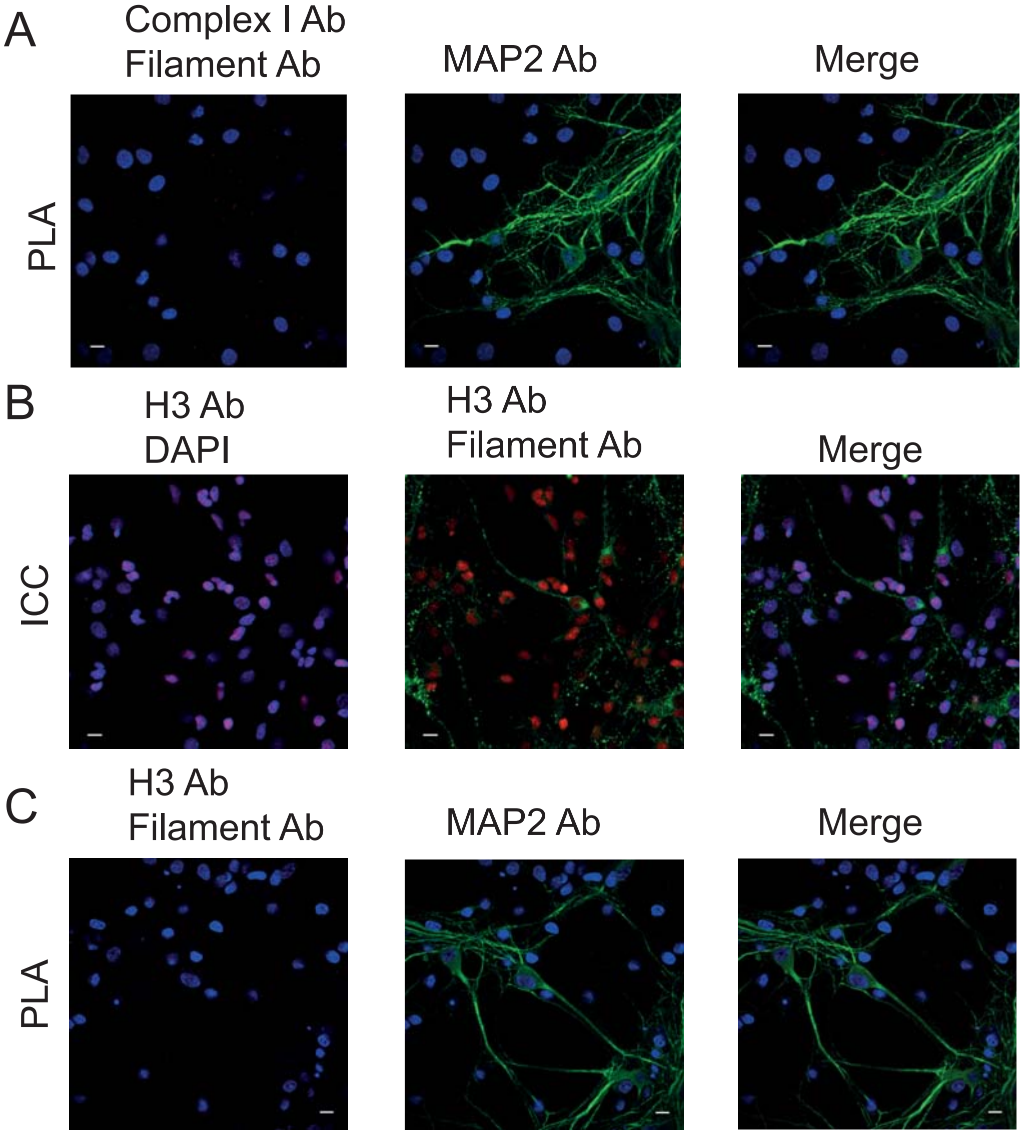

Supplementary figure 3
A) Representative PLA images of cells treated with oligomers probing for Complex I and filament α-synuclein. Cultures were counterstained with the neuronal marker, MAP2. The nucleus can be seen in blue (DAPI). B) Representative ICC images of cells treated with oligomers probing for Histone H3 and filament α-synuclein. Cultures were counterstained with the neuronal marker, MAP2. The nucleus can be seen in blue (DAPI). C) Representative PLA images of cells treated with oligomers probing for Histone H3 and filament α-synuclein. Cultures were counterstained with the neuronal marker, MAP2. The nucleus can be seen in blue (DAPI). Scale bar=10μm

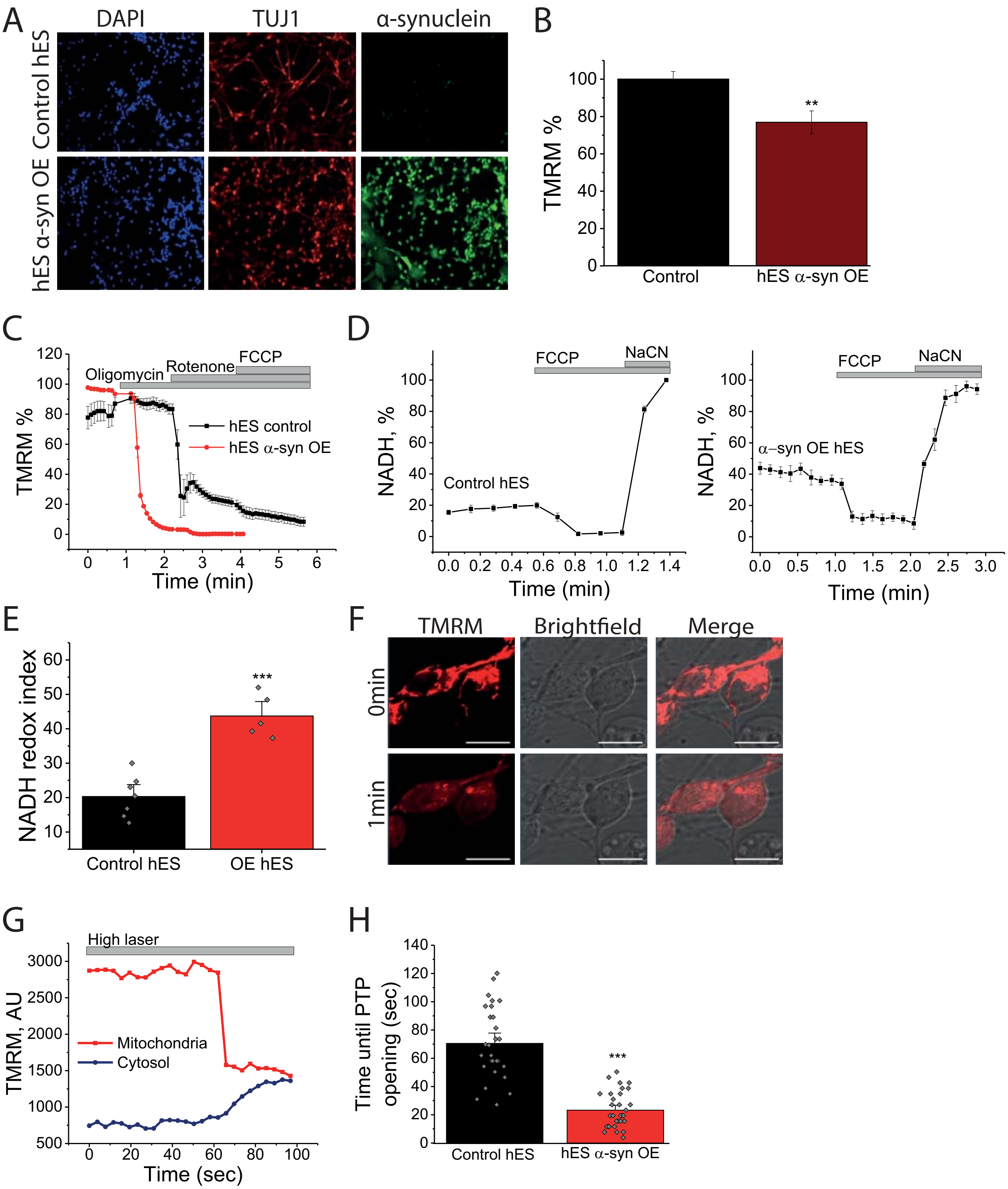

Supplement figure 4

A) Representative ICC images of control and α-syn OE hES probed for TUJ1 and α-synuclein. The nucleus can be seen in blue (DAPI). B) Quantification of the ΔΨm in control and α-synuclein OE hES (control n=12 field of view, OE n=12 field of view, N=3). C) Representative traces of TMRM fluorescence in control and α-synuclein OE hES after addition of oligomycin (2μg/ml), rotenone (1μM), and FCCP (1μM). D) Representative trace of NADH in control and α-synuclein OE hES. FCCP is applied to maximise respiration and therefore minimise the NADH fluorescence signal and NaCN is added to block the mitochondrial respiration and therefore maximise the NADH fluorescence signal. E) Quantification of the redox index in control and α-synuclein OE hES (control n=7; OE n=5; where n=number of cells). F) Representative images of mitochondria labelled with TMRM before high laser power was applied (0min) and after mPTP opening (1min). G) Representative traces of PTP opening in control hES in response to high laser. The red trace shows a sharp drop in TMRM fluorescence intensity upon PTP opening whereas the blue trace represents the leak of TMRM dye into the cytosol upon PTP opening. H) Quantification of the time until PTP opening in response to high laser power (control n=26, OE n=28, N=2). Two-tailed Student's t-test for B and H. Scale bar =10μm. Data represented as ±SEM. **$p<0.01$; ***$p<0.001$

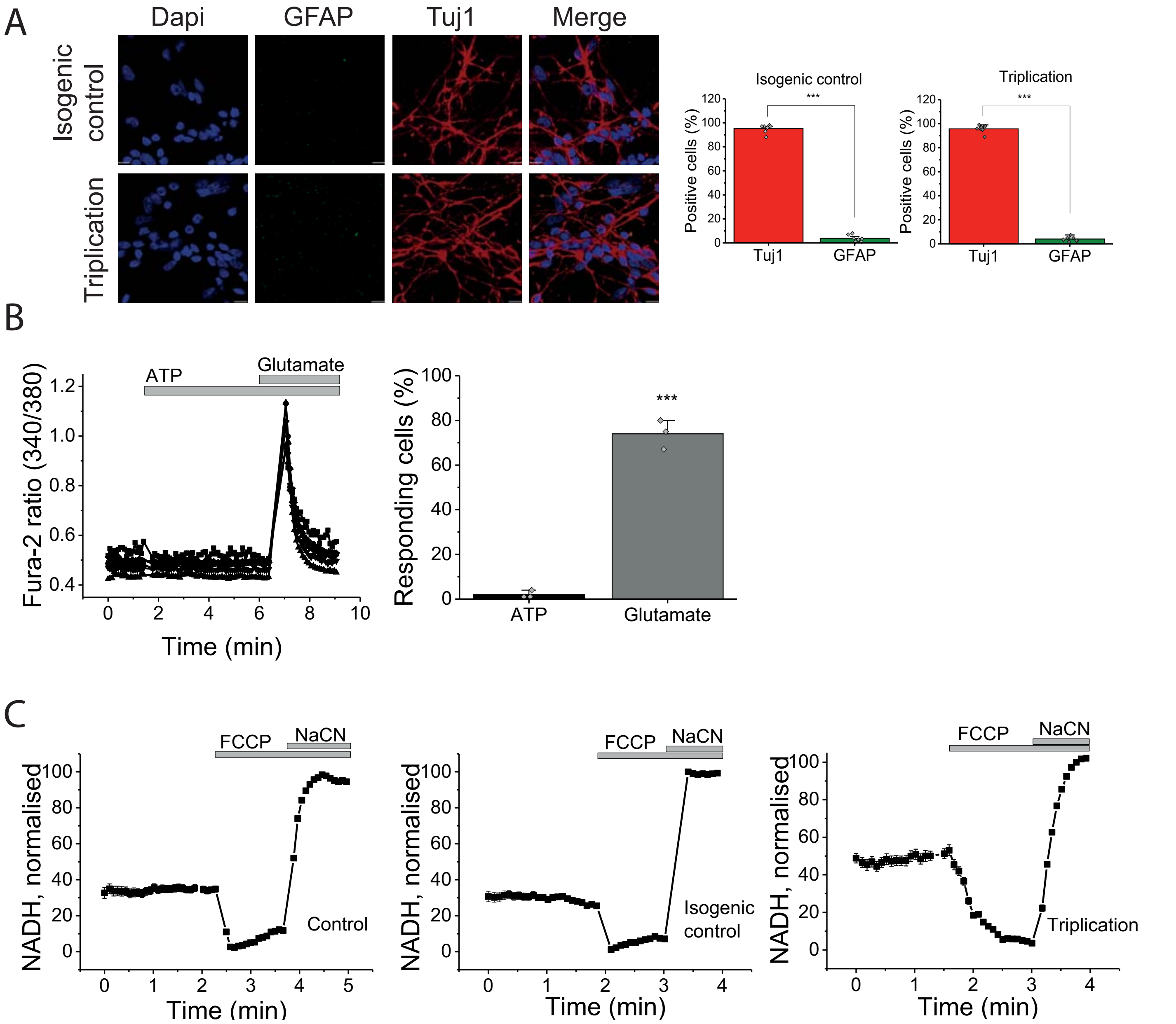

Supplementary figure 5

A) Representative ICC images of the isogenic control and triplication neurons probed for GFAP and TUJ1. The nucleus can be seen in blue (DAPI). Quantification of GFAP and TUJ1 positive cells. B) Representative traces of Fura-2 in control neurons exposed to ATP and glutamate and the quantification of responding cells. C) Representative traces of NADH redox indices for controls (n=166), isogenic controls (n=267) and triplication neurons (n=115); where n=number of cells). 1µM FCCP is applied to maximise respiration and therefore minimise the NADH levels and 1 mM NaCN is added to block the mitochondrial respiration and therefore maximise the NADH levels. Two-tailed Student's t-test for A and B. Data represented as ±SEM. ***$p<0.001$

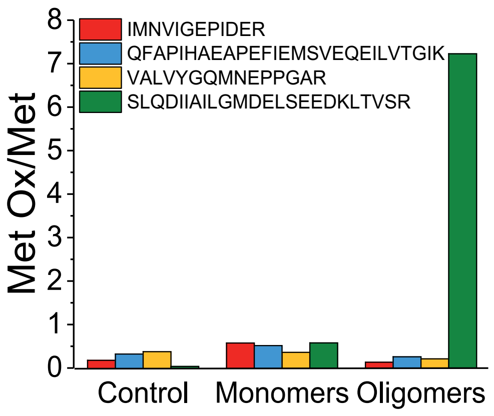

Supplementary figure 6
Ratio of peptides with oxidised methionine versus peptides with non-oxidised methionines ([Met(O)/[Met]).

Supplementary Table 1
Summary of oxidation modifications

Modification	Mass change (Da)	AA
Monooxidation	15.995	C, D, F, H, K, M, N, W, Y
Dioxidation	31.990	C, F, K, M, P, R, W, Y
Trioxidation	47.985	C, W, Y
C-terminal oxidation	15.995	Any
His -> Asn	23.016	H
His -> Asp	22.032	H
Lys -> AminoadipicAcid	14.963	K
